# **An Awkward Fit:** Antimicrobial Resistance and the Evolution of International Health Politics (1945-2022)

**DOI:** 10.1017/jme.2022.78

**Published:** 2022

**Authors:** Claas Kirchhelle, Scott H. Podolsky

**Affiliations:** 1: UNIVERSITY COLLEGE DUBLIN, DUBLIN, IRELAND; 2: HARVARD MEDICAL SCHOOL, BOSTON, MA, USA

**Keywords:** AMR, Antibiotics, Pandemics, International Health Regulations, Sanitary Conferences

## Abstract

Despite being acknowledged as a major global health challenge, growing levels of antimicrobial resistance (AMR) in pathogenic and commensal organisms have proven an awkward fit for international health frameworks. This article surveys the history of attempts to coordinate international responses to AMR alongside the origins and evolution of the current international health regulations (IHR). It argues that AMR, which encompasses a vast range of microbial properties and ecological reservoirs, is an awkward fit for the ‘organismal’ philosophies that centre on the rapid control of individual pathogens that have characterised international policy-making since the 19th century.

## Introduction

The health challenge of antimicrobial resistance (AMR) is as old as the antimicrobial era, with the first known clinical reports of emerging microbial ‘drug fastness’ in response to prolonged organoarsenic exposure dating back to 1907.^1^ Since then, reports of AMR have followed on the heels of the launch of each new class of antimicrobials, from the sulfonamides in the 1930s to lipopeptide antibiotics (daptomycin) in 2003.^2^ The predictable emergence of AMR means that generations of microbiologists, physicians, veterinarians, and policymakers have grappled with how to make the best use of antimicrobials’ time-limited effectiveness. At the international level, at least 248 reports have addressed the issue of AMR since 1945 with topics ranging from ensuring ‘rational’ drug use to addressing the environmental reservoirs of AMR.^3^ Despite the marked increase of AMR reports and initiatives to preserve antimicrobial effectiveness, no binding international agreement on antimicrobial stewardship has emerged. Meanwhile, growing doubts are surfacing about the efficacy of the existing 2015 WHO-led Global Action Plan in galvanizing meaningful national action when it comes to reducing antimicrobial consumption and resistance.^4^

This article examines why AMR has proven so difficult to regulate. Focusing on the period between 1945 and 2015, it contrasts the evolution of AMR stewardship initiatives with the parallel development of international legal frameworks like the International Health Regulations (IHR), which have enjoyed varying levels of success. The article shows that AMR proved an awkward fit for a legal system that was developed around individual pathogens or substances. Traditional contain-and-control strategies developed to stop the spread of well-characterized bacterial and viral entities such as cholera, rinderpest, or influenza.^5^ By contrast, the multitude of organisms and genetic factors involved, the diversity of social and ecological contexts in which AMR arises, the range of potential points for intervention, and the very utility of antibiotics and the competing need for access have to this point rendered AMR ill-suited to the nation-state-oriented matrix of international policy-making. Despite an emerging consensus that AMR can only be tackled by global collective action,^6^ the complex nature of the antibiotic infrastructures embedded in modern health and food production systems,^7^ the dynamic ecological properties of AMR, and the absence of a clearly defined target of action have so far posed an insurmountable obstacle to effective international policy-making.^8^ The article ends by assessing a range of different political and legal approaches towards tackling AMR. It advocates that rather than solely trying to make AMR fit into traditional pathogen-focused policy frameworks, we should aim for a new structural mode of international policymaking, and one that moves beyond conceptualizing AMR solely in terms of present overuse and treatment failure and adopts a more long-term conception of stewarding microbial ecosystems.

Rather than solely trying to make AMR fit into traditional pathogen-focused policy frameworks, we should aim for a new structural mode of international policymaking, and one that moves beyond conceptualizing AMR solely in terms of present overuse and treatment failure and adopts a more long-term conception of stewarding microbial ecosystems.

## From Sanitary Conferences to International Health Regulations

The roots and evolution of our contemporary international health system are intimately tied to the history of pandemics and have consistently focused on containing the spread of individual pathogenic viruses or bacteria across national borders.^9^

Starting in the 19th century, and building on centuries of more local approaches to quarantine, pandemic events like the repeated waves of cholera and plague prompted imperial powers and emerging nation states to organize a total of 14 International Sanitary Conferences. The goal of these conferences was to develop an internationally standardized way of defining, notifying, and reacting to the spread of pathogenic threats. Despite early tensions, the period around 1900 saw the rise of germ-based explanations of disease and mutual concerns about pandemic events’ impact on trade lead to an initial formalization of international health politics in the form of permanent institutions like the *Office International d’Hygiène Publique* (OHIP, 1907-1947) and International Sanitary Conventions (ISCs) (1892, 1903, 1912, 1926).^10^ The explicit aim of these frameworks and institutions was to contain the spread of well-defined pathogens — mostly from Asia to Europe and North America — via timely reporting and appropriate quarantine-focused sanitary interventions, which would be carried out not by international agencies but by nation states. This nation-state and pathogen-focused outlook of the “classical regime”^11^ of international health politics remained mostly unchanged over the next century.

From the 1920s onwards, new permanent international bodies with greater powers like the League of Nations Health Office (LNHO, 1924-1945) and World Health Organisation (WHO 1948-), as well as affiliated national and non-governmental organizations, launched various social medicine-oriented programs (e.g., attempts to improve maternal health and childhood nutrition) alongside vaccination, environmental control, and disease eradication campaigns. However, all of these non-statutory schemes remained dependent on the often unstable political and financial support of sovereign nation states and imperial powers.^12^ Amidst the broader post-1945 decline of concerns about communicable disease, there was also little political appetite to systematically expand or reform the limited statutory reporting and sanitary requirements for pathogenic threats.

In 1951, the International Sanitary Regulations (ISR) consolidated the previous ISC patchwork of reporting regulations for six already quarantinable diseases (cholera, plague, relapsing fever, smallpox, typhoid and yellow fever). Passed in the context of the ‘Hong Kong Flu’ pandemic, the 1969 International Health Regulations (IHR) replaced the ISR but did not include significant new powers. Indeed, reporting requirements were soon limited to three ‘classic’ diseases (cholera, plague, and yellow fever). Meanwhile, critics bemoaned lack of compliance with ISR/IHR provisions and international legal frameworks’ failure to provide means to react to new or re-emerging pathogens such as HIV/AIDS or multiple drug resistant tuberculosis. In practice, the IHRs were thus increasingly overshadowed by the passage of health-relevant international treaties in the fields of human rights, environmental protection, and trade.^13^

Between 1995 and 2005, concerns about pandemic preparedness, bioterrorism, the increasing displacement of international health by trade concerns, and the 2003 SARS outbreak led to sustained attempts to reform the IHRs. Passed in 2005, the revised IHR replaced many classic elements of international disease surveillance with a new concept of integrated biosecurity governance. Although they continued to focus on concerning established pathogens, the 2005 IHR required member states to survey and notify the WHO regarding a far broader range of potential public health risks of urgent international concern — including emergent diseases, intentional bioterrorist releases of disease, and nuclear and chemical hazards. Minimum national surveillance capacities were defined and the WHO could now also use non-governmental data to declare a public health emergency of international concern (PHEIC). In case of a PHEIC, the WHO could issue non-binding recommendations for member states to tackle the emergency, which would theoretically enable a standardized global response.^14^

## AMR: A Non-traditional Threat

Throughout this long history of trying to contain infectious diseases at the international level, the systemic health threat posed by AMR has been curiously absent from statutory health frameworks. Estimated to have caused over 1.27 million human deaths in 2019,^15^ the mortality, morbidity, and economic impact of AMR surpasses that of most individual pathogenic threats identified in both the ISR and subsequent IHR frameworks.^16^ Despite the recent extension of the IHR towards broadly defined ‘public health threats’, the ecological nature of AMR has made it an awkward fit for the organismal- and biosecurity-oriented international health architecture we have inherited.

The fact that AMR or ‘drug fastness’ could emerge as a result of exposing organisms to antimicrobial substances was nothing new by the time the first permanent international health institutions emerged. Concerns about the threat it could pose to new generations of antimicrobial treatments emerged soon after the launch and mass-marketing of sulfonamides (1930s) and antibiotics (1940s) and led to repeated calls for ‘rational therapy.’^17^ However, outside of individual clinical settings, early AMR concerns rarely manifested in concrete regulatory attempts at the national, let alone international level. The reasons for this were manifold: post-war contemporaries struggled to measure and define at which point AMR was not just a ‘natural’ biological phenomenon but a clinical threat; experts (until the 1960s) understood AMR as a localized hereditary rather than an ‘infectious’ ecological phenomenon characterized by horizontal gene transfer; there was no consensus on which forms of antibiotic use were particularly harmful; and there was widespread confidence that antibiotic innovation would stay ahead of AMR.^18^

When they did appear, reform responses to emerging AMR were limited. During the 1950s crisis surrounding the pandemic spread of penicillin resistant *Staphylococcus aureus* phage type 80/81, regulators followed a classic ‘organismal’ control approach aimed at stopping the pathogen with hygiene and updated treatment regimes rather than seeking to limit the evolutionary selection pressure being brought to bear on microbial ecosystems.^19^ In a similar vein, post-war concerns about rising antimicrobial use and emerging AMR in food production were addressed with attempts to limit resistance in a select number of organisms via usage restrictions.^20^

Despite warnings about the ecological impacts of mass antimicrobial usage by prominent microbiologists like gramicidin-discoverer René Dubos,^21^ AMR’s non-standard risk profile meant that regulatory responses remained based on ‘classic’ pathogen-based concepts. Meanwhile, selection pressure continued to increase as a result of falling antimicrobial prices and growing infrastructural dependence on routine antibiotic access. In 1970, alarm about the threat posed by horizontal resistance transfer saw the European Economic Community (EEC) restrict certain forms of therapeutic antibiotic use in agricultural settings. However, bans were partial and measures were not extended to address rising clinical antimicrobial use or antimicrobials’ presence in the environment. Parallel US attempts to implement statutory regulations of human and animal antibiotic use failed.^22^

It was only during the 1980s that sustained calls for wider international efforts to contain AMR emerged. Initially, these calls were voiced not by international organizations but by non-governmental actors. Tufts University clinician-researcher Stuart Levy, especially concerned with resistance-conferring plasmids that respected the boundaries of neither bacterial species nor nation-states, first drew attention to the shared, global nature of AMR. The 1981 *Statement Regarding Worldwide Antibiotic Misuse* that Levy coordinated and had signed by 147 scientists from 27 countries, was multisectoral in its concerns (ranging from the differential marketing and regulation of antibiotics, worldwide, to their use in agribusiness) and was picked up by many press outlets.^23^ Levy’s AMR warnings were soon amplified by Nobelist Joshua Lederberg, who likewise drew attention to the shared moral and pragmatic rationales for focusing on global emerging infections and AMR in parallel.^24^

Yet despite the inclusion of AMR within the U.S. Institute of Medicine’s influential 1992 *Emerging Infections* report,^25^ emphasis on the horizontal epidemiology of AMR-conferring plasmids did not fit easily into the above-mentioned “classic”^26^ pathogen-focused legal regime of international health. Addressing a genetic threat that can jump between pathogens as well as billions of harmless commensal organisms and is both a natural part of bacterial competition and a by-product of antimicrobial infrastructures required significant reductions of selection pressure alongside interventions to detect and counter infectious disease threats. But just how far antimicrobial restrictions would have to go to freeze — let alone reverse — AMR levels remained unclear. It was equally unclear which areas of health and food production systems would have to undertake these cuts. A similar dilemma arose about how to take into account the intergenerational aspects of AMR selection: was it justifiable to ask present generations to forgo drug usage and associated health and efficiency gains for the sake of future generations? Should wealthy areas of the world make larger sacrifices because of their earlier access to and longer large-scale use of antimicrobials than resource-poor areas, who were still struggling to access effective antimicrobials? Without a unifying target, apparent technical fix, or a clear trade-off of sacrifices across different economic sectors and societal groups,^27^ AMR also differed from other contemporary ecological challenges such as the depletion of the ozone layer, species conservation, or marine and cross-border air pollution.^28^

By the end of the millennium, the ongoing rise of AMR levels was increasingly acknowledged as a severe global health threat that spanned all three domains of human, animal, and ecological health.^29^ Driven by reports of MDR tuberculosis, Vancomycin Resistant Enterococci (VRE), and Methicillin Resistant *Staphylococcus aureus* (MRSA), AMR appeared on the policy agendas of powerful nation states and transnational organisations such as the US and the European Union (EU).^30^ However, once again, policy responses — especially when matched against business interests in agribusiness and pharmaceuticals invested in maintaining the *status quo* — were insufficient and tended to follow traditional molds rather than address AMR’s genetic and ecological dimensions. Attempting to fix rather than restructure existing food production and health systems, high-income countries adopted selective usage reductions in human and animal health but mostly neglected the environmental domain whereas new surveillance systems once again focused on resistance in priority pathogens rather than environmental background levels of AMR. Although high-income levels of medical and agricultural usage began to decline by around 2010, interventions’ long-term impact on AMR proved difficult to assess. In some cases, local AMR levels had declined but quickly recovered once drugs were reintroduced; in other cases, interventions came too late to prevent the permanent establishment of AMR in clinical, agricultural, or broader microbial ecologies. Meanwhile, AMR genes and organisms continued to flow freely across borders. As highlighted by reports on *New Delhi metallo-betalactamase 1* (2008) and *mobilised colistin resistance* (2015), addressing AMR required a broader international restructuring of health, food production, and pharmaceutical systems.^31^

The systems-dimension of would-be AMR reform efforts went far beyond anything ever attempted within the conservative legal frameworks of international health. Starting with an ill-fated attempt to galvanise AMR action on September 11, 2001, international organizations such as the WHO, the Food and Agriculture Organisation (FAO), and World Organisation for Animal Health (OIE) began to move from simply reporting on AMR to proposing novel surveillance and stewardship frameworks.^32^ This shift mirrored developments in other areas of Global Health where concerns about bioterrorism and pandemic preparedness had increased donor and member states’ willingness to expand international organisations’ governance competencies.^33^ Unsurprisingly, emerging AMR policy mechanisms thus resembled those created for pandemic preparedness. In 2015, the WHO launched a Global Action Plan (GAP) on AMR.^34^ Similar to the 2005 IHR and the parallel Framework Convention on Tobacco Control, the GAP established parameters for antibiotic stewardship and surveillance that members could attempt to achieve via national action plans (NAPs). NAPs could be monitored via new surveillance mechanisms for AMR and antibiotic usage — but implementation would not be binding. On paper, the GAP marked an important breakthrough, followed in 2016 by the United Nations High-Level Meeting Political Declaration on AMR, intended to further draw global commitment to confronting AMR.^35^ By May 2022, at least 97 nations had developed and submitted NAPs to WHO.^36^

However, similar to problems regarding the 2005 IHR,^37^ creating an international framework based on non-binding stewardship and surveillance recommendations was not the same as investing in actual systems restructuring. Critics of the 2015 GAP soon warned that exporting high-income stewardship approaches that centred on AMR surveillance, reducing usage, and behavioural interventions was ineffective in low- and medium-income settings facing other more pressing health, economic, and governance challenges. With funders prioritizing trackable performance targets, AMR-focused investment ended up favouring technical fixes like antibiotic and diagnostics innovation rather than preventing upstream drivers of AMR such as lacking sanitary infrastructure or affordable health care.^38^ Published in 2019 ahead of the COVID-19 pandemic, a report by the UN Interagency Coordination Group (IACG) on AMR partially acknowledged these challenges by advocating a more robust integration of antibiotic sensitive interventions into existing Sustainable Development Goals (SDGs).^39^

## Conclusion: Quo Vadis AMR

Amidst the wider reordering of Global Health following COVID-19, a growing number of voices are now calling for an integration of the “silent pandemic”^40^ of AMR into a new Pandemic Treaty Framework that would supersede the 2005 IHR by 2024. But what could such an AMR-focused treaty achieve? Pursuing existing policy trajectories, some commentators hope that including the AMR challenge within a statutory international biosecurity framework would overcome the inadequacies of the voluntary GAP. And certain proposed measures — including the harmonization of surveillance measures, the mobilization of resources concerning antimicrobial, vaccine, and diagnostic innovation, and ensuring the equitable distribution of such resources — come closest to fitting traditional frameworks.^41^

In 2000, Lederberg called for a reconsideration of the Manichean “good/bad” mindset regarding bacteria, and for a focus on the broader ecology of the microbial world we inhabit.^43^ While this is not to detract from the immediate need to address the mortality caused by resistant priority pathogens, adopting an ecological approach to mitigating AMR opens a long-term horizon for learning from other planetary challenges.

However, there are well-known limitations. At one level, the described measures do not address the upstream forces — from sanitation to the structures of care delivery — that have differentially shaped the worldwide incidence of AMR and disease burdens. New antibiotics will temporarily reduce AMR pressures, and improved surveillance and infection prevention can address the threat posed by individual pathogens. However, addressing the structural drivers of antibiotic dependency and disease will require far broader ‘antibiotic sensitive’ upstream investment in health and food systems resilience.^42^ Mobilizing such investment during a time of disrupted international relations and economic stress will be challenging — especially if AMR is framed narrowly as a biosecurity challenge of treatment failure. A more holistic additional approach — as Joshua Lederberg attempted to do over three decades ago — lies in stressing the shared moral and pragmatic imperatives to tackle AMR, in this case as a symptom and outcome of larger structural inequities. As such, AMR would represent not just an addition to an existing threat (as has often been the case), but a catalyst for more fundamental change.

Moreover, the invocation of Lederberg calls to mind a second set of considerations. In 2000, and echoing earlier warnings by René Dubos, Lederberg called for a reconsideration of the Manichean “good/bad” mindset regarding bacteria, and for a focus on the broader ecology of the microbial world we inhabit.^43^ While this is not to detract from the immediate need to address the mortality caused by resistant priority pathogens,^44^ adopting an ecological approach to mitigating AMR opens a long-term horizon for learning from other planetary challenges.^45^ In the case of climate change, the international community has acknowledged that the well-being of human societies depends on maintaining global warming within a narrow temperature range. Although far from perfect, resulting international agreements have acknowledged that maintaining this temperature range for future generations requires significant investment by current generations as well as trade-offs between economic sectors and nations. Focusing primarily on the macrobial world, the 1992 Rio Convention on Biological Diversity agreed on the conservation of biodiversity, the sustainable use of its components, and fair and equitable sharing of benefits arising from genetic resources. Could similar legal frameworks be developed for the preservation of the microbial ecospheres humanity relies on?

If AMR signals an “Anthropocene in the cell,”^46^ we should consider extending traditional pathogen-centred regulatory responses to the broader impact of human activity on local and global microbial environments. A rich field of interdisciplinary research surrounding “microbiome stewardship,”^47^ “disaster microbiology,”^48^ “microbial health,”^49^ and “microbial equity”^50^ is already contextualising AMR as a dysbiotic sign of societal and microbial stress. Engaging with emerging insights can help us develop research agendas to orientate global regulatory responses and create equitable infrastructures capable of not only mitigating AMR in the short term, but also of preserving diverse eubiotic microbiota for future generations.^51^ In the long term, addressing AMR as an ecological challenge of microbiome preservation rather than as an awkward addition to classic health diplomacy may prove a more natural fit for this age-old policy challenge.

## Note

Kirchhelle reports funding from the Norwegian Research Council Dry Antibiotic Pipeline project.

## References

[1] C. Gradmann , “Magic Bullets and Moving Targets: Antibiotic Resistance and Experimental Chemotherapy, 1900-1940,” Dynamis 31, no. 2 (2011): 305–321. 22332461 10.4321/s0211-95362011000200003

[2] H. Landecker , “Antimicrobials before Antibiotics: War, Peace, and Disinfectants,” Palgrave Communications 5, no. 1 (2019): 45; T.T. Tran, J.M. Munita, and C.A. Arias, “Mechanisms of Drug Resistance: Daptomycin Resistance,” *Annals of the New York Academy of Sciences* 1354, no. 1 (2015): 32-53. 10.1111/nyas.12948 PMC4966536 26495887

[3] K. Overton , N. Fortané , A. Broom , et al., “Waves of Attention: Patterns and Themes of International Antimicrobial Resistance Reports, 1945–2020,” BMJ Global Health 6, no. 11 (2021): e006909. 10.1136/bmjgh-2021-006909 PMC8573652 34740914

[4] S.R. Van Katwyk , J.M. Grimshaw , M. Nkangu , et al., “Government Policy Interventions to Reduce Human Antimicrobial Use: A Systematic Review and Evidence Map,” PLoS Medicine 16, no. 6 (2019): e1002819; L. Munkholm and O. Rubin, “The Global Governance of Antimicrobial Resistance: A Cross-Country Study of Alignment between the Global Action Plan and National Action Plans,” *Globalization and Health* 16, no. 1 (2020): 1-11; C.D. Iwu and S.M. Patrick, “An Insight into the Implementation of the Global Action Plan on Antimicrobial Resistance in the WHO African Region: A Roadmap for Action,” *International Journal of Antimicrobial Agents* 58, no. 4 (2021): 106411.31185011

[5] F. Vagneron , “Surveiller et s’ unir?. Le rôle de l’OMS dans les premières mobilisations internationales autour d’un réservoir animal de la grippe,” Revue d’anthropologie des connaissances 9, no. 2 (2015): 139–162; M. Harrison, *Contagion: How Commerce Has Spread Disease* (New Haven and London: Yale University Press, 2012); A.K. McVety, *The Rinderpest Campaigns: A Virus, Its Vaccines, and Global Development in the Twentieth Century* (Cambridge: Oxford University Press, 2018).

[6] S.J. Hoffman , G.M. Caleo , N. Daulaire , et al., “Strategies for Achieving Global Collective Action on Antimicrobial Resistance,” Bulletin of the World Health Organization 93 (2015): 867–76. 26668439 10.2471/BLT.15.153171 PMC4669731

[7] C.I. Chandler , “Current Accounts of Antimicrobial Resistance: Stabilisation, Individualisation and Antibiotics as Infrastructure,” *Palgrave Communications* 5, no. 1 (2019): 1–13; C. Kirchhelle, “Pharming Animals: A Global History of Antibiotics in Food Production (1935–2017),” *Palgrave Communications* 4, no. 96 (2018): 96. 10.1057/s41599-019-0263-4 PMC6542671 31157116

[8] C. Kirchhelle , P. Atkinson , and A. Broom , et al., “Setting the Standard: Multidisciplinary Hallmarks for Structural, Equitable and Tracked Antibiotic Policy,” BMJ Global Health 5, no. 9 (2020): e003091. 10.1136/bmjgh-2020-003091 PMC7513567 32967980

[9] See Harrison and McVety, both *supra* note 5; M. Harrison , “Pandemics,” in M. Jackson , ed., The Routledge History of Disease (Abingdon: Routledge, 2017): 129–146.

[10] W.F. Bynum , “Policing Hearts of Darkness: Aspects of the International Sanitary Conferences,” History and Philosophy of the Life Sciences (1993): 421–34; V. Huber, “The Unification of the Globe by Disease? The International Sanitary Conferences on Cholera, 1851–1894,” *The Historical Journal* 49, no. 2 (2006): 453-476; D.P. Fidler, “From International Sanitary Conventions to Global Health Security: The New International Health Regulations,” *Chinese Journal of International Law* 4, no. 2 (2005): 325-92. 7824689

[11] D.P. Fidler , “Emerging Trends in International Law Concerning Global Infectious Disease Control,” Emerging Infectious Diseases 9, no. 3 (2003): 285. 12643821 10.3201/eid0903.020336 PMC2958540

[12] P. Weindling , “Introduction: Constructing International Health between the Wars,” in P. Weindling , ed., *International Health Organisations and Movements, 1918-*1939 (Cambridge Cambridge University Press, 1995): at 1–16; M.D. Dublin, “The League of Nations Health Organisation,” in P. Weindling, ed., *International Health Organisations and Movements, 1918-1939* (Cambridge: Cambridge University Press, 1995): 56-80; P. Weindling, “Social Medicine at the League of Nations Health Organisation and the International Labour Office Compared,” in P. Weindling, ed., *International Health Organisations and Movements, 1918-1939* (Cambridge: Cambridge University Press, 1995): 134-54; M. Cueto, “The Cycles of Eradication: The Rockefeller Foundation and Latin American Public Health, 1918-1940,” in P. Weindling, ed., *International Health Organisations and Movements, 1918-1939* (Cambridge: Cambridge University Press, 1995): 222-244; M. Cueto, T.M. Brown, and E. Fee, *The World Health Organization: A History* (Cambridge: Cambridge University Press, 2019).

[13] See Harrison, *supra* note 5; Fidler, *supra* note 10.

[14] *Id.*

[15] C.J. Murray , K.S. Ikuta , and F. Sharara , et al., “Global Burden of Bacterial Antimicrobial Resistance in 2019: A Systematic Analysis,” Lancet 399 (2022): 629–652. 35065702 10.1016/S0140-6736(21)02724-0 PMC8841637

[16] . *Review on Tackling Drug-Resistant Infections Globally: Final Report and Recommendations* (London: The Review on Antimicrobial Resistance, 2016), *available at* <https://amr-review.org/sites/default/files/160518_Final%20paper_with%20cover.pdf> (last visited Dec. 12, 2022).

[17] . A. Fleming, “Penicillin [Nobel Lecture, December 11, 1945],” *available at* <https://www.nobelprize.org/uploads/2018/06/fleming-lecture.pdf> (last visited Dec. 12, 2022); S.H. Podolsky , *The Antibiotic Era* . Reform, Resistance and the Pursuit of a Rational Therapeutics (Baltimore: Johns Hopkins University Press, 2015).

[18] See Landecker, *supra* note 2; Podolsky, *supra* note 17; S. Podolsky , “The Evolving Response to Antibiotic Resistance (1945-2018),” Palgrave Communications 4, no. 124 (2018); R. Bud, *Penicillin: Triumph and Tragedy* (Oxford: Oxford University Press, 2007); C. Gradmann, “From Lighthouse to Hothouse: Hospital Hygiene, Antibiotics and the Evolution of Infectious Disease, 1950-1990,” *History and Philosophy of the Life Sciences* 40, no. 8 (2017); C. Kirchhelle, *Pyrrhic Progress: Antibiotics in Anglo-American Food Production (1949-2018)* (New Brunswick: Rutgers University Press, 2020); C. Gradmann, “Sensitive Matters: The World Health Organisation and Antibiotic Resistance Testing, 1945-1975,” *Social History of Medicine* 26, no. 3 (2013): 555-574.

[19] K. Hillier , “Babies and Bacteria: Phage Typing, Bacteriologists, and the Birth of Infection Control,” Bulletin of the History of Medicine 80, no. 4 (2006): 733–61; F. Condrau and R.G.W. Kirk, “Negotiating Hospital Infections: The Debate between Ecological Balance and Eradication Strategies in British Hospitals, 1947-1969,” *Dynamis* 31, no. 2 (2011): 385-404. 10.4321/s0211-95362011000200007 PMC3302197 22332465

[20] C. Kirchhelle , “Between Bacteriology and Toxicology - Agricultural Antibiotics and US Risk Regulation,” in A.N.H. Creager and J.-P. Gaudillière , eds., Risk on the Table (New York: Berghahn, 2021).

[21] R.J. Dubos , Mirage of Health: Utopias, Progress and Biological Change, 1959 ed. (New York: Harper & Row, 1959).

[22] See Kirchhelle, *supra* note 7.

[23] See Podolsky, *supra* note 18; “Statement Regarding Worldwide Antibiotic Misuse,” in S.B. Levy, R.C. Clowes, and E.L. Koenig, eds., *Molecular Biology, Pathogenicity, and Ecology of Bacterial Plasmids* (New York: Plenum, 1981): at 679-81.

[24] Podolsky, *supra* note 17, J. Lederberg , “Medical Science, Infectious Disease, and the Unity of Humankind,” JAMA 260, no. 5 (1988): 684–685. 3392795

[25] *Emerging Infections: Microbial Threats to Health in the United States* (Washington, D.C.: Institute of Medicine, 1992). 25121245

[26] See Fidler, *supra* note 10.

[27] See Kirchhelle et al., *supra* note 8; S. R. Van Katwyk, A. Giubilini, C. Kirchhelle, et al., “Exploring Models for an International Legal Agreement on the Global Antimicrobial Commons: Lessons from Climate Agreements,” *Health Care Analysis* (2020): 1-22. 10.1007/s10728-019-00389-3 PMC10042908 31965398

[28] J. Gupta , “A History of International Climate Change Policy,” Wiley Interdisciplinary Reviews: Climate Change 1, no. 5 (2010): 636–53; T. Gehring, *Treaty-Making and Treaty Evolution* (Oxford: Oxford University Press, 2007); S.O. Andersen and K.M. Sarma, *Protecting the Ozone Layer: The United Nations History* (London/New York: Routledge, 2012).

[29] See Overton et al., *supra* note 3.

[30] See Kirchhelle, *supra* note 18.

[31] See Overton et al., *supra* note 3; *Final Report*, supra note 16; S. Davies , Annual Report of the Chief Medical Officer, vol. 2, 2011 *:* Infections and the Rise of Antimicrobial Resistance (London: Department of Health, 2013).

[32] . See Overton et al., *supra* note 3; Kirchhelle, *supra* note 7; Podolsky, *supra* note 18; S.H. Podolsky, R. Bud, C. Gradmann, et al., “History Teaches Us That Confronting Antibiotic Resistance Requires Stronger Global Collective Action,” *Journal of Law, Medicine & Ethics* 43, no. 2, Suppl. (2015): 27-32; N. Fortané “La Surveillance Comme Dispositif-Frontière: La triple ontologie des bactéries résistantes d’origine animale,” *Revue d’anthropologie des connaissances* 9, no. 2 (2015): 265-90.

[33] N.B. King , “The Scale Politics of Emerging Diseases,” Osiris 19 (2004): 62–76; N. Fortané and F. Keck, “How Biosecurity Reframes Animal Surveillance,” *Revue d’anthropologie des connaissances* 9, no. 2 (2015): online; A. Lakoff, *Unprepared: Global Health in a Time of Emergency* (Berkeley: University of California Press, 2017). 15449391 10.1086/649394

[34] WHO, Global Action Plan on Antimicrobial Resistance (Geneva: World Health Organisation, 2015).

[35] *Political Declaration of the High-Level Meeting of the General Assembly on Antimicrobial Resistance* (New York: UN General Assembly, 2016).

[36] WHO, Library of AMR National Action Plans (Geneva: World Health Organisation), *available at* <https://www.who.int/teams/surveillance-prevention-control-AMR/national-action-plan-monitoring-evaluation/library-of-national-action-plans> (last visited Dec. 12, 2022).

[37] H. Kluge , J.M. Martín-Moreno , N. Emiroglu , et al., “Strengthening Global Health Security by Embedding the International Health Regulations Requirements into National Health Systems,” BMJ Global Health 3, no. Suppl. 1 (2018): e000656. 10.1136/bmjgh-2017-000656 PMC5783036 29379650

[38] See Overton et al., *supra* note 3; Munkholm and Oliver, *supra* note 4; Kirchhelle et al., *supra* note 8; S. Nayiga , L. D. Willis , S.G. Staedke , and C.I. Chandler , “Reconciling Imperatives: Clinical Guidelines, Antibiotic Prescribing and the Enactment of Good Care in Lower-Level Health Facilities in Tororo, Uganda,” Global Public Health (2022): 1–12, doi: 10.1080/17441692.2022.2045619; A. Doron and A. Broom, “The Spectre of Superbugs: Waste, Structural Violence and Antimicrobial Resistance in India,” *Worldwide Waste: Journal of Interdisciplinary Studies* 2, no. 1 (2019), https://doi.org/10.5334/wwwj.2; L.D. Willis and C. Chandler, “Quick Fix for Care, Productivity, Hygiene and Inequality: Reframing the Entrenched Problem of Antibiotic Overuse,” *BMJ Global Health* 4, no. 4 (2019): e001590.

[39] IACG, *No Time to Wait: Securing the Future from Drug-Resistant Infections*, New York Interagency Coordination Group on Antimicrobial Resistance, April 2019, *available at* <https://www.who.int/docs/default-source/documents/no-time-to-wait-securing-the-future-from-drug-resistant-infections-en.pdf> (last visited Dec. 12, 2022).

[40] A.R. Mahoney , M.M. Safaee , W.M. Wuest , and A.L. Furst , “The Silent Pandemic: Emergent Antibiotic Resistances Following the Global Response to SARS-CoV-2,” Iscience 24, no. 4 (2021): 102304. 33748695 10.1016/j.isci.2021.102304 PMC7955580

[41] L.A. Wilson , S.R. Van Katwyk , I. Weldon , and S.J. Hoffman , “A Global Pandemic Treaty Must Address Antimicrobial Resistance,” Journal of Law, Medicine & Ethics 49, no. 4 (2021): 688–91. 10.1017/jme.2021.94 PMC8749967 35006051

[42] See Kirchhelle et al., *supra* note 8; K. Thornber and C. Kirchhelle , “Hardwiring Antimicrobial Resistance Mitigation into Global Policy,” JAC-Antimicrobial Resistance 4, no. 4 (2022): dlac083. 10.1093/jacamr/dlac083 PMC9345311 35928475

[43] R.J. Dubos , Man Adapting (New Haven: Yale University Press, 1985); J. Lederberg, “Infectious History,” *Science* 288 (2000): 287-93; J. Lederberg, “Beyond the Genome,” *Brooklyn Law Review* 67 (2001): 7-12.

[44] See Murray et al., *supra* note 15.

[45] See Thornber and Kirchhelle, *supra* note 42.

[46] F. D’Abramo and H. Landecker, “Anthropocene in the Cell,” *Technosphere Magazine* (2019): 1-7.

[47] R.S. Peixoto , C.R. Voolstra , M. Sweet , et al., “Harnessing the Microbiome to Prevent Global Biodiversity Loss,” Nature Microbiology 7 (2022): 1726–1735. 10.1038/s41564-022-01173-1 35864220

[48] D.F.Q. Smith and A. Casadevall , “Disaster Microbiology: A New Field Of Study,” MBIO 13, no. 4 (2022): e01680–22. 35920557 10.1128/mbio.01680-22 PMC9426413

[49] C. Kirchhelle and A.P. Roberts , “Embracing the Monsters: Moving from Infection Control to Microbial Management,” Lancet Microbe 3, no. 11 (2022): E806–807. 35964637 10.1016/S2666-5247(22)00225-7

[50] S.L. Ishaq , M. Rapp , R. Byerly , et al., “Framing the Discussion of Microorganisms as a Facet of Social Equity in Human Health,” PLoS biology 17, no. 11 (2019): e3000536; S.L. Ishaq, F.J. Parada, P.G. Wolf, et al., “Introducing the Microbes and Social Equity Working Group: Considering the Microbial Components of Social, Environmental, and Health Justice,” *MSystems* 6, no. 4 (2021): e00471-21. 31770370 10.1371/journal.pbio.3000536 PMC6879114

[51] G. Berg , D. Rybakova , D. Fischer , et al., “Microbiome Definition Re-visited: Old Concepts and New Challenges. Microbiome 8, no. 1 (2020): 1–22. 32605663 10.1186/s40168-020-00875-0 PMC7329523

